# Behavioral Functionality of Mobile Apps in Health Interventions: A Systematic Review of the Literature

**DOI:** 10.2196/mhealth.3335

**Published:** 2015-02-26

**Authors:** Hannah E Payne, Cameron Lister, Joshua H West, Jay M Bernhardt

**Affiliations:** ^1^Computational Health Science Research GroupDepartment of Health ScienceBrigham Young UniversityProvo, UTUnited States; ^2^Center for Health CommunicationUniversity of TexasAustin, TXUnited States

**Keywords:** smartphone, app, health behavior, systematic review, interventions

## Abstract

**Background:**

Several thousand mobile phone apps are available to download to mobile phones for health and fitness. Mobile phones may provide a unique means of administering health interventions to populations.

**Objective:**

The purpose of this systematic review was to systematically search and describe the literature on mobile apps used in health behavior interventions, describe the behavioral features and focus of health apps, and to evaluate the potential of apps to disseminate health behavior interventions.

**Methods:**

We conducted a review of the literature in September 2014 using key search terms in several relevant scientific journal databases. Only English articles pertaining to health interventions using mobile phone apps were included in the final sample.

**Results:**

The 24 studies identified for this review were primarily feasibility and pilot studies of mobile apps with small sample sizes. All studies were informed by behavioral theories or strategies, with self-monitoring as the most common construct. Acceptability of mobile phone apps was high among mobile phone users.

**Conclusions:**

The lack of large sample studies using mobile phone apps may signal a need for additional studies on the potential use of mobile apps to assist individuals in changing their health behaviors. Of these studies, there is early evidence that apps are well received by users. Based on available research, mobile apps may be considered a feasible and acceptable means of administering health interventions, but a greater number of studies and more rigorous research and evaluations are needed to determine efficacy and establish evidence for best practices.

## Introduction

Since 2007, mobile phones like Apple’s iPhone and Google’s Android have taken over the mobile market; 56% of Americans now own a smartphone [1]. Third-party apps are software programs that serve to expand the utility of mobile devices. Within just 6 years, Apple celebrated its 50 billionth app download with Google trailing only slightly behind with 48 billion as of May 2013 [2]. This new market of software apps has resulted in over $9 billion being paid to developers for Apple alone [2]. Health apps have also become a part of this market, with over 31,000 health and medical apps available for download [3]. With mobile phone ownership and the number and complexity of health apps likely to increase, the potential for technology-based health interventions to impact populations is possible like never before.

Health-related apps now number more than 31,000 [3], and their utility for promoting health behavior has been analyzed [3-5]. Most published literature regarding health apps has focused on preventing and managing chronic disease [6,7], monitoring health behaviors and vitals [8], content analyses of health and fitness apps [9-11], app acceptability and utility [12-15], and qualitative studies of user experience and desired functions [4,16,17]. However, most studies are descriptive. Recently, several published studies have utilized apps in health behavior interventions; however, it is unclear how substantial this body of literature currently is, or the extent to which mobile apps are shown to be efficacious at facilitating behavior change. The purpose of this systematic review was to describe the current body of literature on apps used in health behavior interventions, describe the behavioral features and focus of apps, and evaluate the potential of apps to efficaciously disseminate health behavior interventions.

## Methods

A search of published, peer-reviewed literature was conducted in September 2014 for articles that studied health behavior interventions that utilized mobile apps. The researchers used key search terms to identify potential articles (see Table 1). We queried JMIR, Academic Search Premier, CINAHL, Communication and Mass Media Complete, Computer Source, Computers and Applied Sciences Complete, Health Source—Consumer Edition, Health Source—Nursing/Academic Edition, Medline, PubMed, PsychARTICLES, Psychology and Behavioral Sciences Collection, Web of Science, and PsycINFO. Only articles published after 2007 were considered because that was the year the first app-ready mobile phone entered the market.

**Table 1:** Search terms for systematic review.

Search lines	Search terms	Filtered by
Line 1	Smartphone OR mobile phone OR Mobile device* OR tablet OR iphone OR “mobile technolog*” OR “Smart Phone” OR ipad OR mhealth OR android OR windows	Title/ Abstract
2. AND	App OR apps OR “mobile app” OR application*	Title/Abstract
3. AND	health OR BMI OR “heart disease” OR “physical activity” OR diabetes OR smoking OR exercise OR cancer OR obesity OR nutrition OR “public health”	Title/ Abstract
4. AND	behavior OR behaviour OR intervention OR “controlled trial” OR RCT	Title/ Abstract
5. NOT	developing OR telemedicine OR “text messaging” OR SMS	Title

**Figure 1:**
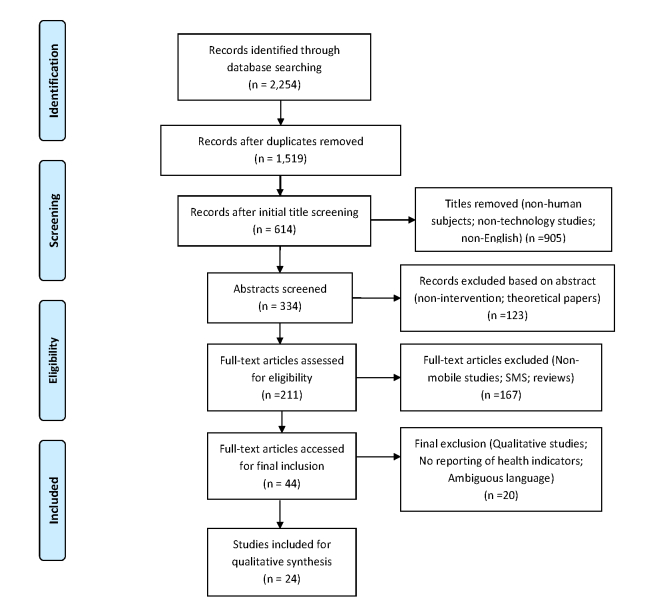
Systematic review of the literature flowchart.

Our query returned 2254 articles. The authors reviewed the titles of articles and abstracts and eliminated duplicates and studies of non-human subjects, which reduced the sample to 334 articles. Further inclusion and exclusion criteria were applied to the sample of articles. Inclusion criteria included using a mobile app (iPhone, Android, or Windows); an intervention study of some type; use of behavioral theory, constructs, or strategies; studying a public health topic with health indicators reported; and published after 2007 to the time of the search (September 19, 2014). Studies were excluded if they were non-English studies, if they assessed an app through qualitative data only, if they had ambiguous language pertaining to whether or not they used a mobile phone app, or if they used a Web-based app and not specifically a mobile phone app. This resulted in a final sample of 24 articles selected for inclusion in the current systematic review (see Figure 1) [12,18-40].

## Results

### Overview

Seventeen of the studies had a sample of less than 100 participants. Gajecki et al [24] had the largest sample (N=1929) and was a randomized multi-group trial. Eleven of the studies reviewed were feasibility or pilot tests, and the majority of the studies used a randomized study design (15 studies). Post-intervention follow-up assessment for the 9 studies ranged between 4 weeks and 6 months, with the exception of Quinn et al [32] and Mattila et al [31], where the follow-up periods extended to 1 year. Eighteen of the studies tested an app that had been developed specifically for the intervention, while the remaining five tested existing apps. The majority of studies (18) reported testing one app. Hebden et al [26] tested four, which was the most in any study (see Multimedia Appendix 1).

Of the studies reviewed, 14 involved interventions for physical activity and diet, four studies involved diabetes management, four for improving mental health, and only two studies involved interventions for addiction.

### Behavioral Components of Mobile Apps

All of the studies incorporated at least one prominent health behavior theory construct or strategy. Self-monitoring was the most common, included in 18 of the studies. The next most commonly used constructs were cues to action and feedback (both included in nine studies each), followed by social support (six studies). Major theories used as frameworks included social cognitive theory (four studies) and self-determination theory (two studies).

Every app used for mental health and addiction was either designed with a specific behavioral strategy or selected with a behavioral construct in mind (eg, the theory of planned behavior, self-determination theory, behavioral activation approach) (100%, 6/6), and all but one of the physical activity and diet interventions were constructed after a specific construct or theory (93%, 13/14). The diabetes apps were the least likely to be designed or chosen with specific behavioral constructs in mind (25%, 1/4), or rather, the apps selected happened to include behavioral constructs.

### User Retention and Acceptability

The mean retention rate for smartphone use throughout the intervention period was 79.6%, with a low of 29% and a high of 100%. Retention rate for these studies was defined as the number of initial study participants who remained in the study through the intervention period and follow-up. Of the studies that reported on user acceptability (13 studies), most reported high user acceptability and feasibility of using smartphone apps for behavior change interventions, except Gajecki et al [24] and Matilla et al [31] who reported average and low-to-moderate acceptability, respectively.

Of the diet and physical activity apps, Allen et al [19] reported that users were most satisfied with the accountability and structure of the app and the use of a smartphone platform. Users suggested a stronger emphasis on exercise and additional feedback. Bond et al [20] reported that real-time smartphone display and feedback significantly increased their motivation to engage in physical activity. Users in the Brindal et al [21] study indicated that most found the app easy to use and that weight tracking and prompting were the most popular feature, while trophies and meal graphs (visual presentation) were the least. Burns et al [22] indicated that users felt the intervention was helpful in understanding negative mood triggers and managing distressing behaviors and thoughts. Receiving the mood predictions was the most helpful component of the intervention, and many suggested a verbal component when using the smartphone. Carter et al [40] reported that users found the apps convenient and easier to use than other methods of tracking diet. Additionally, users felt more comfortable using the smartphone app in public.

Matilla et al [31] utilized three separate apps and found that users most enjoyed the ability to see progress over time, having a record of personal progress and seeing development in long-term health outcomes, and adaptive exercises and coaching. Unexpectedly, some users noted that they did not like being pressured by the apps to do healthy activities and viewed it as a barrier to use. King et al [27] reported that the main acceptable features of the apps developed for the study were ease of use, limited time per use, and high acceptability of awareness raising and alerts to action. Robinson et al [12] reported similar findings, with users reporting high acceptability of ease of use and convenience of the app. Users reported acceptability of more automatic functions, and an app that worked into their daily routine. Additionally, users reported acceptability of awareness raising of behaviors and wanted discrete interactions with the app in public. Smith et al [33] reported that users particularly enjoyed the “push prompt” used in the app and that teacher satisfaction with the app was particularly high. Thomas et al [34] reported that almost all users indicated maximum rating for both satisfaction with the app and likelihood in recommending it to others. Finally, Kirwan et al [28] also reported that the most important/acceptable components of the app were ease of use and time, with the average time of use being 9.3 seconds.

Only one of the diabetes studies reported acceptability; Cafazzo et al [23] reported that users were more satisfied with using the app for diabetes monitoring, and the interactions with the app were considered “fast”. Additionally, users wanted the interactions to be discrete in public to avoid embarrassment in social settings. Rewards and sharing of information with family members was also rated high in acceptability. Similarly, only one of the addiction studies reported details concerning acceptability. Gajecki et al [24] did not report specifics about acceptability or user opinion and only assessed how easy the app was to use, how suitable it was for helping those with risky alcohol consumption, and whether participants would recommend it to friends. On all three, scores were average (3.2-3.6 on a 5-point scale).

### Efficacy of Mobile App Interventions

Ten studies targeted physical activity as a primary measure, and eight of them reported increases. Allen et al [19], King et al [27], Mattila et al [31], Turner-McGrievy et al [35], Turner-McGrievy et al [36], and Van Drongelen et al [37] measured physical activity from either self-reporting on their apps or through questionnaires, and all but Turner-McGrievy et al [35] reported increases in physical activity, with Allen et al [19] reporting only a slight increase. Bond et al [20], Hebden et al [26], Kirwan et al [29], and Smith et al [33] utilized objective measures, either from the apps themselves or from accelerometers. All but Smith et al [33] reported significant increases in physical activity.

Ten studies targeted Body Mass Index (BMI) or weight loss, and all but one reported either decreases in BMI, weight loss, or decreases in body fat except one. Allen et al [19], Brindal et al [21], Carter et al [40], and Hebden et al [26] all reported higher amounts of weight loss or lower BMIs in the smartphone interventions, but the weight loss was not statistically significant, including when compared to controls. Mattila et al [31] noted that weight, body fat, and BMI all decreased, and there was a significant difference between sustained app users and non-sustained users. Robinson et al [12] noted decreases in body weight, but it was a secondary measure and few details of the weight loss were reported. Smith et al [33] reported no significant decreases in BMI or body fat. Thomas et al [34] reported significant decreases in body weight at 12-week follow-up, but not at 24 weeks. Turner-McGrievy et al [35,36] reported no significant difference in weight loss between intervention groups in the 2011 study, while in the 2013 study, users experienced a significant drop in BMI at follow-up. Of the four interventions related to diabetes, three reported a positive change in glycated hemoglobin: Kirwan et al [29] and Quinn et al [32] showed a significant decrease in HbA1c levels, while Wayne et al [39] showed a significant decrease only for those whose baseline HbA1c levels were above 7%. Cafazzo et al [23] reported no change.

The three mental health interventions that addressed depression reported significantly decreased depression levels at follow-up, while the last one, a stress management intervention (Ahtinen et al [18]), reported significantly better stress ratings and life satisfaction at follow-up. Of the two apps addressing addiction, one showed a significant improvement in number of risky drinking days, while the other reported no effect on alcohol consumption, with one app possibly showing a negative effect on alcohol consumption.

It is also worth noting that many studies (Hebden et al [26], Mattila et al [31], both Turner-McGrievy studies [35,36], and Quinn et al [32]) utilized apps as part of a comprehensive intervention strategy; that is, the interventions were not specifically designed for a smartphone, and the apps were used as part of a multicomponent strategy.

## Discussion

### Principal Findings

Despite the thousands of health and fitness apps now available for download and the emerging interest in using them for improving health behaviors, very few have been tested in intervention settings. This lack of evaluation may be concerning because smartphone owners have an average of 41 apps installed [41], with 52% using their phones for health purposes and 19% using health apps [42]. Additionally, the types of published, peer-reviewed app studies that were available for review were predominantly small sample pilot or feasibility studies, instead of more rigorous randomized controlled trials (RCTs) with adequately powered samples. Notwithstanding the small number and nature of the studies included in this review, the majority of evaluated apps were found to be inclusive of health behavior theory constructs such as self-monitoring and goal-setting. The existing literature does illustrate high user acceptability of smartphone apps for health interventions and shows preliminary potential of these apps to change behaviors and, subsequently, health outcomes.

### Behavioral Components of Mobile Apps

Constructs from Bandura’s Social Cognitive Theory (SCT) were the most used in the reviewed studies as is evidenced by the presence of self-monitoring and social support, both prominent in SCT [43]. Other studies and articles have focused on the accuracy of self-monitoring with mobile technology [8], a trend that is consistent with the Quantified Self-Movement [44]. Additionally, social support was emphasized in several of the studies reviewed. In both her studies, Turner-McGrievy et al [35,36] utilized Twitter as a supplementary tool for participants to share tips and comments with one another. Cafazzo et al [23] utilized a similar tool, allowing for participants to interact on a social network with parents, peers, and clinical staff to report progress. King et al [27] divided participants into groups to interact online so each group could support one another and see each other’s progress. Wayne et al [39] provided health coaches as additional support and personal counseling to help participants adhere to behavioral goals. Social support has been shown to be useful for increasing motivation or providing reinforcement for changes in behavior [45,46].

### App Acceptability and Efficacy

The findings of this review may demonstrate the potential of using apps to increase retention in health behavior interventions. These findings also mirror larger societal trends wherein consumer acceptance and demand for health and fitness apps to change behavior is growing [3], which has resulted in increased profitability [2].

Several of the articles reported improvements in physical activity. For example, King et al [27] reported that participants increased physical activity and decreased sedentary time, Kirwan et al [28] reported that use of an app was associated with greater number of steps logged, and van Drongelen et al [37] reported that users increased the number of days they experienced both moderate and strenuous intensity exercise each week. In other research, a content analysis of 127 physical activity apps for behavior change theory showed that most apps had low behavior change potential and recommended that developers partner with experts in behavior change to increase app quality [9]. Many of the apps reviewed in this study were developed specifically for the purposes of their respective interventions, which may account for their effectiveness for increasing physical activity. This raises questions about the potential for effecting behavior change between existing apps and apps developed for a specific intervention.

Ten interventions reviewed in this study reported a change in weight loss or reduction in BMI or body fat, but only three (Mattila et al [31], Turner-McGrievy et al [36], and Thomas et al [34]) reported significant results, and Thomas et al [34] only reported significant results at the first follow-up, with significance diminishing after further study. The results of this review point to significant potential barriers in the use of health apps as interventions for weight loss. This should be studied further in light of recent qualitative research that has identified barriers to using calorie counting apps for diet change, mostly due to the complexity of calorie counting apps [47]. Additional research of weight-loss apps has also been completed concluding that many apps have low evidence-informed content, demonstrating lack of industry standards and inclusion of evidence-based practices [48].

The diabetes studies showed conflicting results. Cafazzo et al [23] reported no measurable change in HbA1c, although glucose monitoring did increase during the intervention period. After a 1-year follow-up, Quinn et al [32] found improvements among participants’ glycated hemoglobin. However, previous reviews of self-management diabetes apps report that apps may not be adequate for providing comprehensive self-management care and adhering to clinical guidelines [49,50]. Further research is needed to determine the benefits of app interventions for diabetes management.

Watts et al [38] measured depression and psychological distress among individuals diagnosed with major depression. There were no measurable differences between the intervention and control group, but depression was lowered among participants using the smartphone app. The current literature is lacking in the area of mobile technology interventions and mental health. The number of mental health apps available for download is also low [51], and future research in the field of mental health should focus on developing and integrating mobile technology into providing treatment for mental disorders.

For developers and future researchers, several of the findings of this study on acceptability of certain components of apps may be found useful. For instance, many of the studies found that users want apps that are fast and easy to use and that allow for discrete interactions in public, with many users reporting being socially conscientious of writing down or reporting personal data in public. Additionally, users reported high acceptability of apps that raised awareness of certain behaviors and provided potential cues to action. Finally, developers and researchers may find promise in integrating rewards into the interventions with smartphone apps to drive better behavioral outcomes.

### Limitations and Future Directions

The inclusion and exclusion criteria were developed in order to capture the most relevant studies involving mobile apps in behavioral interventions to impact health. However, there was no distinction made between studies that used a mobile app in one arm of a multi-group study, or if they were used as the principle focus of the study. Our aim was to not attempt to interpret the original study author’s intentions and to be more inclusive of studies involving mobile apps. Additionally, due to the dearth of apps that met the aims of this systematic review, it was beneficial to be more inclusive to better reflect the current literature. It should be noted that it may be difficult to compare these types of studies. Future systematic reviews may be able to be more restrictive once more studies begin to appear in the academic literature that are more robust and concrete in purpose.

Key future directions are recommended based on the findings of this study. The majority of these studies were pilot or feasibility studies with small samples. Considering the capacity of mobile technology to offer interventions to populations at minimal cost, this finding was surprising. Additionally, with the app industry extending into the billions of dollars, it is concerning that more money is not being put into researching the efficacy of these apps on a large scale. Several apps available for download in the Apple App and Google Play stores have several thousands of customer reviews. Some app companies boast hundreds of thousands of consistent users as well. In the future, health researchers should partner with successful app companies and producers in studying the efficacy of apps to impact health behavior on a much larger scale.

### Conclusions

The purpose of this systematic review was to provide a description of app-based intervention studies, describe common behavioral features, and explore the acceptability and potential for apps to change behavior as currently dictated by the literature. In the small sample of reviewed studies, the majority of apps were viewed as acceptable, inclusive of theory, and efficacious at changing behavior. Moreover, the potential for scalable behavioral interventions through these technologies is promising, but largely untapped. Moving forward, researchers should focus on conducting rigorous RCT studies with adequately powered sample sizes to determine the utility of app-based health interventions. Future researchers may also focus on the potential benefits to behavior change when multiple apps are combined together in one single intervention.

## Multimedia Appendix 1

Characteristics of eligible studies.

## Abbreviations

BMI: Body Mass Index
RCT: randomized controlled trial
SCT: Social Cognitive Theory
