# Supplementary material for: Behavioral Functionality of Mobile Apps in Health Interventions: A Systematic Review of the Literature
Source: JMIR Mhealth Uhealth. 2015 Feb 26;3(1):e20. doi: 10.2196/mhealth.3335 (PMC4376122; doi:10.2196/mhealth.3335)
Supplement: Supplementary file 1 [file mhealth_v3i1e20_app1.pdf]

Multimedia Appendix 1. Characteristics of eligible studies.

Author	Study sample	Intervention Time-table	Study design	Health indicators measured	Behavioral constructs	App(s) used	Retention rate	Results
Ahtinen, Aino[2013] [19]	N=15	1 month	Field Study	Stress, psychological flexibility, and life satisfaction	General information, self-monitoring, reinforcement, feedback	[1]Oiva	No mention	Improvements in stress rating and satisfaction with life scores, no significant improvement in psychological flexibility.
Allen, Jerilyn K.[2013] [20]	N=68, BMI 28-42	6 months	Pilot study, RCT	Exercise, Food Intake, Weight	SCT, Self-management, motivational interviewing	[2]Lose It!	63%, 100% acceptability	Counseling plus app saw the greatest weight loss, app alone saw the least. Slight increase in physical activity in app group.
Bond, Dale S.[2014] [21]	N=30, BMI >25	4 weeks	Experimental trial	Sedentary time, physical activity	Self-monitoring, goal-setting, reinforcement, cues to action	[1]B-Mobile app, with armband sensor	85.7%, 90% acceptability	Percent time in physical activity was significantly increased, and percent time in sedentary activity was significantly decreased from baseline.
Brindal, Emily[2013] [22]	N=58, women, BMI 26-43	8 weeks	Small group randomized trial	Weight loss	Cues to action, self-monitoring, support and feedback	[1]Support app	75.8%; 95% acceptability of app	Greater weight loss was seen by the intervention group; however the difference between the groups was not

								statistically significant.
Burns, Michelle N.[2011] [23]	N=8; diagnosis of major depressive disorder	8 weeks	Single-arm field trial	Depressive symptoms, diagnostic criteria for Major Depressive Disorder	Behavioral activation approach, behavioral coping strategies, cues to action, stress management, emotional coping strategies	[1]Mobilyze!	87.5%; high satisfaction (5.71 on a 7-point scale)	Significant improvement on depression scores, less likely to meet criteria for major depressive disorder. Comorbid anxiety symptoms also decreased.
Cafazzo, Joseph A. [2012] [24]	N=20, aged 12-16	12 weeks	Pilot study,	A1C, monitoring of glucose	Gamification, social support, cues to action, self-monitoring	[1]Bant App for Apple	60%; 88% Acceptability	High satisfaction of using the app for monitoring. Increased glucose monitoring by 50%. A1C did not change.
Carter, Michelle C. [2013] [53]	N=128, aged 18-65, >27BMI	6 weeks, and 6 month follow-up	Pilot study, RCT, 86.8% Acceptability	Dietary recording, BMI*, body fat	Goal setting, self-monitoring, feedback, self-efficacy	[1]My Meal Mate (MMM)	93% in smartphone group; 86.8% Acceptability	Adherence, weight loss, BMI reduction, and reduction in body fat were highest in smartphone group.
Gajecki, Mikael[2014] [25]	N=1929, Mean age=24.720, high alcohol consumption	7 weeks	Randomized multi-group design	Measures of alcohol consumption (binge drinking, frequency, eBAC, etc)	TPB*, self-monitoring, feedback, cues to action, negative affect management	[2]Check Your BAC;	70.6%; average acceptability	eBAC calculation showed no effect on alcohol consumption. One app may have shown a negative effect on alcohol consumption for men

Gustafson, David[2014] [26]	N=349, Mean age=38.3, alcohol dependant (DSM-IV)	8 months, 4 month follow-up	Unmasked randomized clinical trial	Risky drinking days (number of days during which patient's drinking in a 2-hour period exceeded 4-standard drinks for men, 3 for women)	Self-determination theory, social support, self-monitoring, ecological momentary assessment, negative affect management	[1]A-CHESS (Addiction-Comprehensive Health Enhancement Support System)	100%	Patients using the app reported significantly fewer risky drinking days (mean 1.39 vs 2.75 in control group)
Hebden, L.[2013] [27]	N=51, BMI 24-31.99	12 weeks	Pilot study, RCT	BMI, physical activity, sitting time, fruits and vegetables	Self-monitoring, cues to action, feedback	[1]Four applications developed to monitor behaviors	90.1%	Body weight decreased among both the intervention and control group. No significant difference between intervention and control.
King, Abby C. [2013] [28]	N=68, aged 45+, 73% women	8 weeks	Feasibility testing	Changes in PA*, sitting time	*SCT, Social support, self-monitoring, feedback, goal-setting, reinforcement	[1]Three apps: analytical, social, affective	89.7%; 87% reported easy to use	Significant increases in mean physical activity levels, and significant decreases in sitting time, high acceptability of using the apps.
Kirwan, Morwenna [2012] [29]	N=50 treatment, N=150 Control	3 months	Matched Case-Control	Number of steps	Self-monitoring	[1]iStepLog Application	100%; 89% acceptability	Use of the app was associated with greater steps logged; app use was also greater than web use. High usefulness and usability scores for app.

Kirwan, Morwenna[2013] [30]	N=72, Mean age=35.20, diabetic	6 months, 3 month follow-up	RCT	Glycemic control (HbA1c)	Self-monitoring	[2]Glucose Buddy	74%	App users reported decreased HbA1c levels than those in the control group. No significant change over time in self-efficacy, self-care activities, or quality of life in either group.
Ly, Kien Hoa[2014] [31]	N=81; Suffering from major depression (DSM IV)	8 weeks	Paired RCT	Depression symptoms (BDI and PHQ-9)	Cues to action, self-monitoring, reinforcement, feedback, general information	[1]Behavioural Activation App<br>[1]Mindfulness App	70%;	Both apps demonstrated a reduction in depression symptoms. Behavioral Activation app was more effective than the Mindfulness App for those with higher baseline depression levels, while the Mindfulness App was more effective for those with mild baseline depression.
Mattila, Elina[2013] [32]	N=352, ave. 45 yrs, BMI 27-34	1 year	RCT	Weight, PA, sleep, stress, smoking, alcohol consumption	TTM*, self-monitoring, stress management	[2]The Wellness Diary, Mobile Coach, SelfRelax	29%; low to moderate acceptability	Sustained mobile app users realized better weight management results upon follow-up, in addition to decreased aerobic fitness, blood pressure, and total

								cholesterol.
Quinn, Charlene C.[2011] [33]	N=213	1 year	Cluster-randomized trial	Glycated hemoglobin levels, diabetes symptoms	Motivational support, general information	[1]Mobile-phone app for diabetes coaching	76.5%	Mobile phone group did have better measures of glycated hemoglobin after 1 year follow-up
Robinson, Eric[2013] [12]	N=12, BMI >25	4 weeks	Feasibility testing	Calorie consumption, weight	Raising awareness, self-monitoring,	[1]Calorie counting app	100%; high accept-ability	Most found the app easy to use, intention to use was high. Mean weight loss was 1.5kg.
Smith, Jordan J.[2014] [34]	N=361, aged 12-14 boys	20 week	Cluster RCT	BMI, waist circumference, body fat %, PA, screen-time	Self-determination, SCT, skills training, self-efficacy, goal-setting, self-monitoring	[1]ATLAS app	81.1%; 44% enjoyed the app; high teacher acceptability	No significant impact on BMI, or body composition measures. Intervention effects were found for screen-time, sugar-sweetened beverages, and muscular fitness.
Thomas, J Graham[2013] [35]	N=20, BMI 25-50	12-24 weeks	Pilot study	Weight, BMI	Self-monitoring, feedback, skills training	[1]Health-E-Call application	75%; high satisfaction	After 24 weeks of intervention all participants lost 5% of body weight, with 87% losing 10%.
Turner-McGrievy, Gabrielle [2011] [36]	N=96, aged 18-60, BMI >25	3 months, and 6 month follow-up	Randomized multi-group weight-loss intervention	Weight loss, diet and PA monitoring	SCT, self-monitoring, social support	[2]FatSecret Calorie Counting	No mention	Weight loss did not differ between groups. No difference in fat intake or weight-related eating behaviors. More podcasts were downloaded by

								smartphones.
Turner-McGrievy, Gabrielle[2013] [37]	N=96, 18-60, BMI >25	6 months	Randomized weight-loss trial	Dietary outcomes, PA, BMI	Self-monitoring, social support	[2]Run-Keeper, FatSecret, My fitness pal	80-90%	PA app users monitored more often and had a lower BMI and more weight loss upon follow-up.
Van Drongelen, Alwin [2014] [38]	N=502, airline pilots	6 months	RCT	Fatigue, sleep quality, strenuous PA, snacking	General information, feedback, cues to action	[1]MORE Energy app	79.2%	Intervention group saw statistically significant improvements in fatigue, sleep quality, strenuous PA, and snacking.
Watts, Sarah[2013] [39]	N=35, major depression	3 months	Pilot study, RCT,	Depression, psycholo-gical distress	*CBT, vicarious learning, self-efficacy,	[1]Get Happy Program App	65.7%	Depression levels and psychological distress was lowered in both groups, with no significant difference between groups.
Wayne, Noah[2014] [40]	N=21, Mean age=55.6, Type 2 diabetes and HbA1c<9.5%	6 months	Single-arm Longitudinal Feasibility	HbA1c levels	Self-monitoring, cues to action, social support, coaching	[1]Health Coach	100%	Patients demonstrated a mean reduction of 0.34% in Hba1c levels.

Notes:

[1] Indicates that the smartphone app that was used for this study was developed for the purpose of the study by the research team.

[2] Indicates that the smartphone app was already in existence and was merely used for the purposes of the study.

*SCT – Social Cognitive Theory

*TTM – Trans-theoretical Model

*TPB – Theory of Planned Behavior

*PA – Physical Activity

*BMI – Body Mass Index
*CBT – Cognitive Behavioral Therapy
